# Decoding the therapeutic mechanism of *Conocarpus lancifolius* in hepatocellular carcinoma: network pharmacology, molecular docking, and LC-MS QTOF insights

**DOI:** 10.3389/fphar.2025.1582374

**Published:** 2025-06-17

**Authors:** Poojaben M. Prajapati, Saumya K. Patel, Rakesh M. Rawal, Komal G. Lakhani, Rasmieh Hamid, Bharat B. Maitreya

**Affiliations:** ^1^ Department of Botany, Bioinformatics and Climate Change Impacts Management, School of Sciences, Gujarat University, Ahmedabad, Gujarat, India; ^2^ Department of Medical Biotechnology, Gujarat Biotechnology University, Gandhinagar, Gujarat, India; ^3^ Department of Biotechnology, College of Agriculture, Junagadh Agricultural University, Junagadh, Gujarat, India; ^4^ Hawkesbury Institute for the Environment (HIE) at Western Sydney University (WSU), Richmond, NSW, Australia; ^5^ Department of Plant Breeding, Cotton Research Institute of Iran (CRII), Agricultural Research, Education, and Extension Organization (AREEO), Gorgan, Iran

**Keywords:** *C. lancifolius*, LCMS QToF, molecular docking, molecular dynamic simulation, network pharmacology

## Abstract

Hepatocellular carcinoma is a multifaceted and lethal malignancy, ranking third in cancer-related mortality and sixth in worldwide incidence. This study aimed to utilize LCMS-QTOF analysis to identify the phytoconstituents of *C. lancifolius* across three distinct seasons. The study also sought to elucidate the multi-layered mechanism of action against hepatocellular carcinoma using network pharmacology analysis, molecular docking, and molecular dynamics simulation. A total of 352 phytoconstituents were identified in the extract of *Conocarpus lancifolius*, of which 154 compounds were chosen for subsequent *in silico* analysis. Network construction and Gene Ontology (GO) enrichment analysis were performed using ShinyGo and the KEGG database, while Cytoscape 3.10.2 was employed for network visualization and analysis. Molecular docking analyses were conducted using YASARA software, and the highest-scoring compounds and targets underwent 100 ns molecular dynamics simulations via Schrödinger Desmond. CytoHubba identified ten key hub genes, including CASP3, STAT3, and EGFR. GO and KEGG analyses revealed significant biological processes, molecular functions, cellular components, and pathways, including PPAR signaling, thyroid cancer, and prolactin pathways. Notably, phytochemicals from *C. lancifolius*, particularly Alnusiin, Egrosine, and Yessotoxin, exhibited strong binding affinities with CASP3 and STAT3. The structural stability of Alnusiin in complex with these target proteins was confirmed through molecular dynamics simulation, indicating its potential as a promising anti-HCC agent. This study integrates network pharmacology, molecular docking, and molecular dynamics simulations to characterize the bioactive compounds in *C. lancifolius* and elucidate a plausible mechanism for its therapeutic action against hepatocellular carcinoma.

## Graphical Abstract

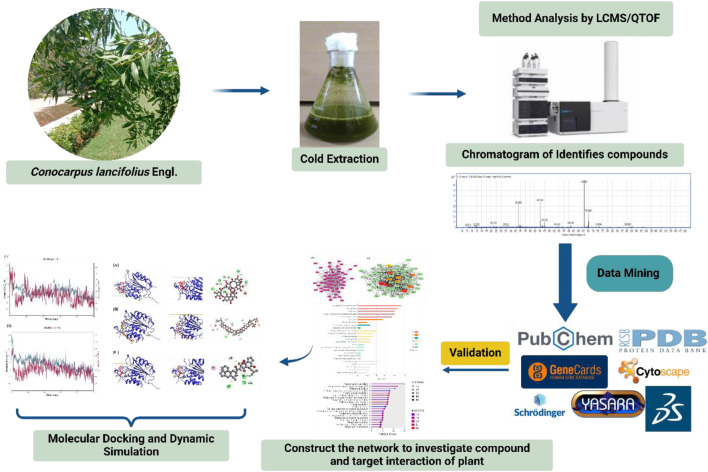

## Highlights

• Identified 352 phytoconstituents in *Conocarpus lancifolius* by LCMS-QTOF analysis, with 154 compounds chosen for *in silico* investigations.
• Network pharmacology and molecular docking identified crucial hub genes, including CASP3, STAT3, and EGFR, as well as significant pathways.
• Alnusiin exhibited an excellent binding affinity to the CASP3 and STAT3 targets.
• Molecular dynamics simulations demonstrated the stability of Alnusiin, indicating its potential as an anti-hepatocellular carcinoma agent.
• Proposed a multi-tiered mechanism for the therapeutic action of *C. lancifolius* against hepatocellular carcinoma.

## 1 Introduction

Hepatocellular carcinoma (HCC) is the most common form of primary liver cancer, accounting for 70%–85% of cases worldwide (Magalhães et al., 2023). HCC typically arises from preexisting liver conditions, including chronic infections caused by the hepatitis B virus (HBV) or hepatitis C virus (HCV), as well as cirrhosis (Suresh et al., 2020). In its early stages, HCC often remains asymptomatic, posing significant challenges for accurate diagnosis and timely intervention. Consequently, HCC has a high mortality rate, emphasizing the need for early detection and effective preventive strategies to improve clinical outcomes and reduce the global burden of liver cancer (Qu et al., 2019).

Various therapeutic strategies for HCC include sorafenib, liver transplantation, transarterial chemoembolization, local ablation, and surgery (Llovet et al., 2015). Sorafenib, a multi-kinase inhibitor targeting RAF, VEGFR, and PDGFR, remains the main pharmacological option for advanced HCC but offers limited efficacy and is poorly tolerated by most patients (Mazzoccoli et al., 2016; Li et al., 2015). This highlights the urgent need for more effective and less toxic treatment alternatives to improve patient outcomes. Chinese herbal medicine (CHM), with its extensive historical application, presents a promising strategy for hepatocellular carcinoma (HCC) treatment and is a significant resource for the development of alternative anticancer medicines (Yang et al., 2017; Wang et al., 2014). Research indicates that some phytochemicals found in medicinal plants have the ability to stop tumour growth, stop cell division, and stop metastases (Yan et al., 2017; Zhang et al., 2017). These substances frequently exhibit more selectivity for cancer cells than traditional chemotherapy, minimising damage to healthy tissues and making them appealing options for cancer treatment (Mazzoccoli et al., 2016).

Medicinal plants contain a variety of secondary metabolites, including anthocyanins, terpenoids, phenolics, flavonoids, polyphenols, alkaloids, and carotenoids, which play significant roles in their therapeutic effects and disease prevention. Bioactive compounds are extensively utilised in pharmaceutical research, acting as significant sources for the discovery and development of novel drugs (Policegoudra et al., 2012). The Combretaceae family, recognised for its therapeutic properties, comprises approximately 20 species, with Combretum and Terminalia being intensively researched for their phytochemical and pharmacological activities (Saadullah et al., 2020). There are two species in the *Conocarpus* genus: *Conocarpus lancifolius* and *Conocarpus erectus*. Native to East Africa and portions of Pakistan, *C. lancifolius* is a hardy ornamental plant that has long been used to cure conditions like skin ulcers, fever, diabetes, diarrhoea, and catarrh (Baroon and Razzaque, 2012). Studies have reported its antibacterial (Raza et al., 2020; Ali et al., 2013), antidiabetic, antioxidant, anti-urease, phytotoxic, cytotoxic, anti-inflammatory, and PPAR-agonistic activities (Saadullah et al., 2016). Additional studies have shown that chloroform leaf extracts of *C. lancifolius* exhibit significant antioxidant and anti-inflammatory activities. Since chronic inflammation and oxidative stress are major drivers of liver carcinogenesis, these bioactivities suggest that *C. lancifolius* may have potential as a supportive agent in the prevention or treatment of hepatocellular carcinoma (HCC) (Parthiban et al., 2023).

The purpose of this study is to determine the active ingredients, targets, and multi-target pharmacological mechanisms that underlie *C. lancifolius* Engl.'s anti-HCC capability. We investigated the intricate relationships between bioactive substances and target proteins using an integrated network pharmacology and bioinformatics methodology. Following the validation of anticipated compound–target interactions using molecular docking, molecular dynamics simulations were used to assess the stability and conformational dynamics of docked complexes over a period of 100 nanoseconds. This is the first thorough analysis of *C. lancifolius’s* effectiveness and mechanisms against HCV-associated HCC, offering important theoretical insights and laying the groundwork for further experimental and clinical studies.

## 2 Methods and materials

### 2.1 Collection of plant material

Leaf samples of the *C. lancifolius* Engl. plant were collected from Ahmedabad, Gujarat, throughout three distinct seasons: summer, winter, and rainy. Dr. Bharat Maitreya, a taxonomist from the Department of Botany, Bioinformatics, and Climate Change Impacts Management at Gujarat University, identified and authenticated the obtained samples.

### 2.2 Extraction

The foliage of the selected plant was separated and washed thoroughly with running tap water to remove any dust particles. After air-drying for several days, the leaves were ground into a fine powder and stored in polythene bags for future use. A total of 10 g of plant material was extracted with 100 mL of acetone solvent using a standard solvent extraction method. The extraction process was carried out at room temperature for 24 h. The resulting extract was then passed through Whatman filter paper No. 1 to separate the solvent and obtain the dehydrated extract.

### 2.3 LCMS QTOF

The LC-MS QTOF analysis was performed to identify the components present in the *C. lancifolius* leaf extract collected during three different seasons under optimized conditions. The LC-MS QTOF system was equipped with a photodiode array (PDA) detector, and a symmetric ZORBAX 300SB C-18 column (4.6 × 100 mm, 3.5-micron particle size) was used for the initial identification of compounds. Ionization was performed in both positive and negative electrospray ionization (ESI) modes. The analysis was carried out using formic acid and water as mobile phases in varying concentrations. The mass spectrometry conditions were set to detect compounds within a mass-to-charge (m/z) range of 80–1,500. The desolvation temperature was maintained at 350°C, with the column and sample temperatures set to 40°C and 15°C, respectively. The system was operated at a flow rate of 0.6 mL/min with an injection volume of 10 µL. The capillary voltage was set at 4000 V, and the desolvation gas flow rate was maintained at 12 L/min. To identify the bioactive compounds, the UNIFI software and a scientific library were utilized.

### 2.4 Network pharmacology-based analysis

The phytoconstituents identified through LC-MS QTOF analysis were subjected to network pharmacology-based investigations. The three-dimensional (3D) molecular structures of the identified compounds were uploaded to PharmMapper, an online server that employs the pharmacophore mapping method to identify potential therapeutic targets (Liu et al., 2010; Bisht et al., 2024). In this study, multiple potential target genes were identified for each compound using PharmMapper.

### 2.5 Targets prediction for HCC

The HCC-associated target genes were gathered from the GeneCards database (https://www.genecards.org/) (Safran et al., 2010). GeneCards is a comprehensive, searchable platform that integrates information on all known and predicted human genes. The database was queried using the keyword “Hepatocellular Carcinoma,” and the most relevant genes were selected based on predefined score thresholds to identify the most relevant disease-related targets.

### 2.6 Gene ontology and KEGGs pathway enrichment

Gene Ontology (GO) and KEGG pathway enrichment analyses were conducted to uncover the biological relevance of the overlapping target genes identified in this study. These analyses enabled the prioritization of genes involved in key pathways and processes directly related to the disease mechanism under investigation. Functional annotation was performed using the DAVID tool, with significance set at p < 0.05 (Dennis et al., 2003). To better interpret the findings, the most enriched GO terms and KEGG pathways were visualized through the KOBAS web server and ShinyGo platform, highlighting critical biological processes, molecular functions, and cellular components specifically implicated in the study’s context (Xie et al., 2011).

### 2.7 Protein-protein network

A Protein-Protein Interaction (PPI) network of the overlapping target genes was constructed using Cytoscape 3.9.1 software (NIGMS, Bethesda, MD, United States) (Paul et al., 1971). Within the graphical network framework, each bioactive compound is represented as a node, with interactions depicted as edges. Molecular network properties were analyzed using the Cytoscape Network Analyzer plugin. Composite interaction scores from Cytoscape were utilized to assess node significance, while the CytoHubba plugin (Chin et al., 2009) prioritized targets and compounds based on their MCC values, identifying key hub nodes for further investigation. A compound–gene interaction network was constructed to visualize the relationships between compounds and their target genes.

### 2.8 Molecular docking analysis

#### 2.8.1 Ligand collection and preparation

Chemical compounds from C. *lancifolius* leaves were obtained through LC-MS QTOF analysis, generating a library of potent bioactive molecules. The molecular structures of these compounds were retrieved from PubChem (Kim et al., 2023). The structures were then processed for molecular docking analysis using Maestro 11.1’s Ligand Preparation wizard, applying the OPLS_3 force field (Chen et al., 2016).

#### 2.8.2 Protein preparation

A literature-based selection was conducted to identify the most relevant target proteins among the top hub nodes. CASP3 and STAT3 (PDB ID: 1XKK and 6TLC, respectively) were chosen for docking studies. Protein structures were prepared using YASARA Structure software (version 19.12.14) (Ozvoldik et al., 2023) through the following steps: (a) Optimizing the binding site, (b) Applying Kolman corrections, (c) Calculating Gasteiger charges, (d) Removing heteroatoms and water molecules, (e) Restoring missing C-terminal oxygen atoms, and (f) Adding hydrogen atoms to ensure stability (Lakhani et al., 2025).

The optimization process prioritized hydrogen bond formation while minimizing steric clashes. The AMBER 14 force field was applied to refine protein structures, and the SiteMap program was used to assess protein quality and active site suitability.

#### 2.8.3 Docking analysis

To evaluate the interaction strength between selected ligands and target proteins, molecular docking was performed using YASARA Structure (Lakhani et al., 2024). Ligands were pre-processed using the “clean” module, and docking was conducted with the AMBER 14 force field to ensure accurate pose prediction. Binding affinities were assessed based on docking scores, with lower energy values indicating stronger interactions (Aamir et al., 2018). Visual inspection of binding conformations was carried out using BIOVIA Discovery Studio Visualizer v4.5 to confirm interaction stability and relevance to the study’s therapeutic focus (Studio, 2008). Top-performing compounds, selected for their strong receptor engagement, were further analyzed through molecular dynamics simulations.

### 2.9 Molecular dynamic simulation

To assess the stability and dynamic behaviour of high-affinity ligand–protein complexes relevant to HCC, 100 ns molecular dynamics (MD) simulations were performed using Schrödinger Maestro. Simulations were also conducted on the corresponding apo-proteins to provide a comparative baseline. The Desmond module, with the OPLS3 force field and explicit solvent conditions, was employed to model realistic biological environments. The simulation was performed in the NPT ensemble at a constant temperature of 300 K and a pressure of 1.01325 bar, maintained for 1 μs to ensure system equilibrium. Prior to this, energy minimization was carried out using the steepest descent method with the OPLS3 force field (Banks et al., 2009). Structural stability and flexibility of the complexes were evaluated through RMSD and RMSF analyses, offering critical insights into the conformational integrity and binding persistence of key therapeutic candidates (Ahammad et al., 2021).

## 3 Results

### 3.1 LCMS QTOF

The total ion chromatogram of the acetone extract of C. *lancifolius* leaves, obtained from LC-MS QTOF analysis, provides a comprehensive profile of the detected phytochemicals. Supplementary Table S1 presents the complete inventory of compounds identified in this study. Using this high-resolution analytical technique, we identified 104 phytochemicals in the summer season, 117 in the rainy season, and 131 in the winter season. These phytochemicals belong to various metabolic families, including phenolic acids (e.g., hydroxybenzoic acid derivatives), flavonoids (e.g., flavanols and fatty acids), and saponins (e.g., triterpene and steroid glycosides). In the chromatograms for the rainy, winter, and summer seasons, the alnusiin compound consistently showed the highest score across all samples, with only minor variations in peak area due to seasonal differences. Although these seasonal changes slightly affected the area under the curve, the overall prominence of alnusiin remained stable across seasons. The identification of Yessotoxin in *C. lancifolius* leaves through LC-MS analysis is a remarkable finding that invites further exploration into the plant’s biochemical processes. While Yessotoxin is commonly associated with marine organisms, particularly shellfish, its presence in a terrestrial plant suggests potential environmental or ecological interactions that might lead to its accumulation. Given that *C. lancifolius* is found in coastal areas, it is plausible that marine toxins could be absorbed through the plant’s interactions with its surrounding environment, such as atmospheric deposition, soil, or water enriched with marine-derived compounds. Moreover, this discovery may indicate the presence of previously unrecognized biosynthetic capabilities within *C. lancifolius*, possibly involving marine microorganisms that produce Yessotoxin. Certain terrestrial plants have been shown to harbour symbiotic relationships with microbes capable of synthesizing marine toxins, suggesting a potential mechanism for the plant’s acquisition of such compounds. Therefore, the identification of Yessotoxin in *C. lancifolius* underscores the importance of further research to better understand the plant’s ecological interactions and the mechanisms behind its ability to integrate marine biotoxins into its metabolic pathways.

Compound identification was conducted based on retention times (RT), UV spectra, molecular weight, and the mass-to-charge ratio (m/z) of molecular ions [M-H]-. This identification was further supported by accurate mass analysis from MS and MS/MS spectra in Supplementary Material S2, spectral database comparisons, and empirical formula generation based on detected peaks. After removing duplicate entries, a total of 154 distinct phytochemicals were selected for subsequent *in silico* investigations.

For the docking studies, a rigorous selection process was employed to refine the initial pool of 352 phytochemicals identified from the LC-MS analysis of *C. lancifolius* leaves. A total of 154 compounds were selected based on several critical criteria aimed at ensuring their biological relevance and computational suitability. Initially, only compounds with well-defined and structurally unambiguous profiles were chosen, eliminating those with incomplete or unclear structures. This step was essential to ensure the integrity of the molecular representations used in the docking simulations. In addition to structural clarity, only those compounds with documented or potential bioactivity related to hepatocellular carcinoma (HCC) were prioritized. This was determined through a comprehensive literature review focused on compounds known to interact with pathways involved in cancer progression, particularly those relevant to HCC. To ensure the compounds’ pharmacological potential. Notably, sulfur-containing compounds were excluded from the selection process. The exclusion of these compounds was due to their potential to introduce significant computational challenges in molecular docking, as sulfur atoms can form strong covalent bonds with metals or other sulfur-containing groups, leading to complex interactions that are difficult to predict accurately. Furthermore, many sulfur-containing compounds may have bioactivities unrelated to the targeted pathways, which could reduce the specificity of the docking results. A target prediction analysis using PharmMapper was performed, retaining only those compounds with a >80% matching degree to known targets. This approach ensured that the selected compounds were likely to interact with proteins involved in HCC, optimizing the focus of the study on therapeutically relevant compounds. This multi-step filtering process, a refined set of 154 compounds was chosen for subsequent docking studies, enhancing the relevance, accuracy, and computational feasibility of the predicted interactions.

### 3.2 Network pharmacology from LCMS QTOF compounds

#### 3.2.1 Target prediction and PPI analysis

PharmMapper is an online tool that predicts potential drug-target interactions by comparing inverted pharmacophore patterns against an internal pharmacophore model database (Shawky, 2019). Using this approach, a total of 65 genes were identified as potential targets for *C. lancifolius* compounds. The Protein-Protein Interaction (PPI) data obtained from STRING was imported into Cytoscape 3.10.2 for network analysis.

**FIGURE 1:**
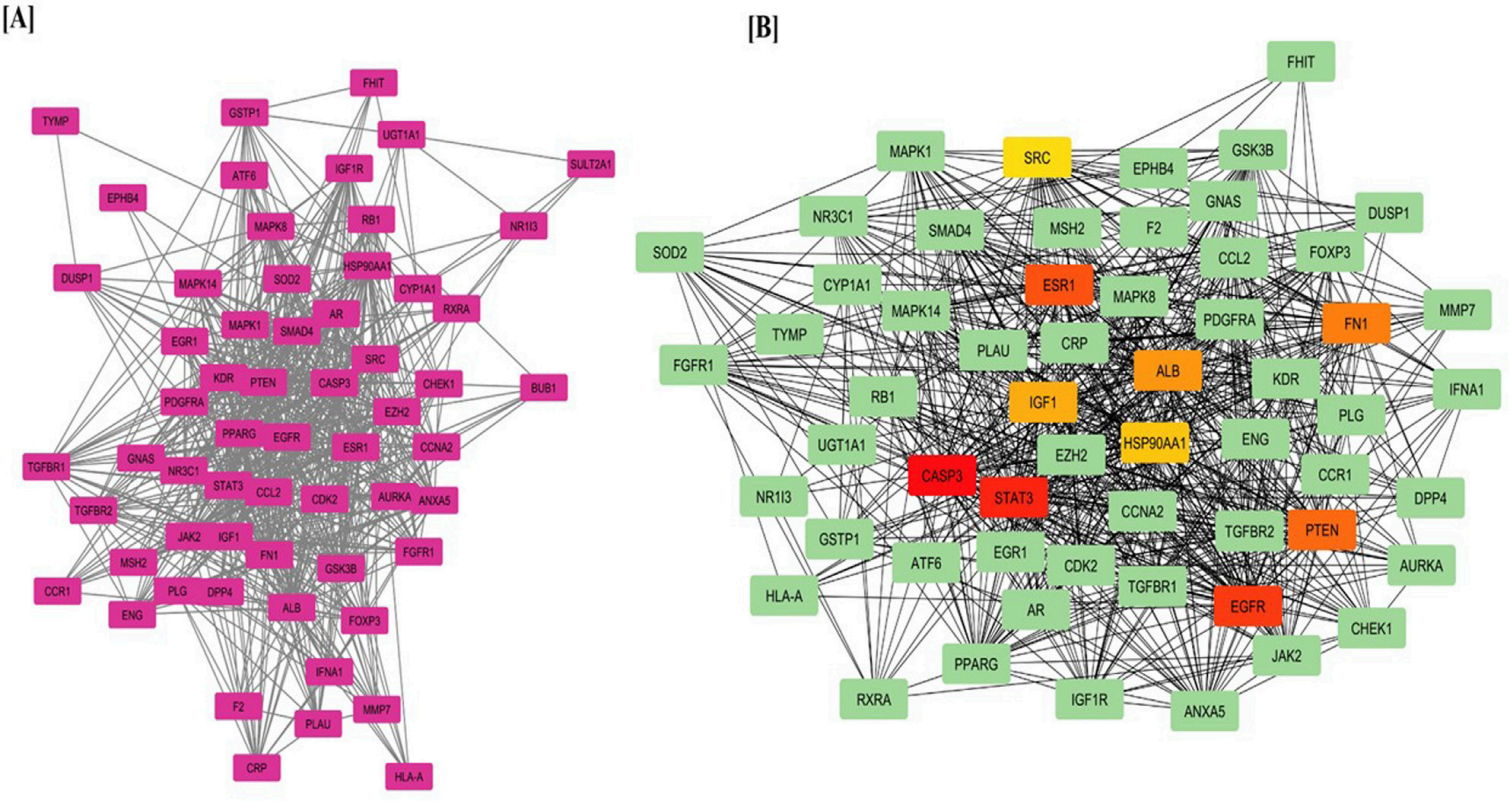
**(A)** Protein–Protein Interaction (PPI) network of target genes predicted from LCMS-QTOF-identified compounds and **(B)** Top hub genes identified using the MCC algorithm via the CytoHubba plugin in Cytoscape.

The mean degree of all nodes was calculated as five. Consequently, 65 target genes with a degree value of five or higher were selected for further analysis. Figure 1A illustrates the PPI network for the 65 selected target genes. Nodes with higher degrees are represented in more intense colors, while node diameters and edge thicknesses correspond to Betweenness Centrality and Edge Betweenness values, respectively. The PPI analysis incorporated multiple significance metrics, including Maximum Neighborhood Component (MNC), Degree, Bottleneck, Closeness, Radiality, Betweenness, and Stress. The Maximum Clique Centrality (MCC) algorithm was selected for hub gene identification due to its superior sensitivity in detecting highly connected and functionally significant nodes within complex biological networks. Unlike simpler topological metrics such as degree or betweenness centrality, MCC identifies nodes that participate in multiple cliques, which are indicative of densely interconnected subgraphs often corresponding to core functional modules. Previous comparative studies have shown that MCC outperforms other centrality measures in predicting biologically meaningful hub genes, particularly in disease-specific networks. Therefore, MCC was employed as the primary algorithm in this study to enhance the robustness of hub gene selection. However, network topology was also visually examined to ensure consistency with biological expectations shows in Table 1.

**TABLE 1:** Top hub nodes calculated through topological analysis MCC methods from LCMS QTOF compound of *C. lancifolius* plant.

MCC	MNC	Degree	EPC	BottelNeck	Closeness	Radiality	Stress
CASP3	EGFR	EGFR	EGFR	CASP3	EGFR	EGFR	EGFR
STAT3	STAT3	STAT3	STAT3	ALB	STAT3	STAT3	STAT3
EGFR	ALB	ALB	CASP3	STAT3	ALB	ALB	ALB
ESR1	CASP3	CASP3	PTEN	PDGFRA	CASP3	CASP3	HSP90AA1
PTEN	ESR1	ESR1	ALB	ESR1	ESR1	ESR1	ESR1
FN1	PTEN	HSP90AA1	HSP90AA1	IGF1	HSP90AA1	HSP90AA1	CASP3
ALB	HSP90AA1	PTEN	ESR1	HSP90AA1	PTEN	PTEN	PTEN
IGF1	SRC	SRC	SRC	EZH2	SRC	SRC	SRC
HSP90AA1	IGF	IGF1	IGF1	CDK2	IGF1	IGF1	PPARG
SRC	PPARG	PPARG	FN1	AR	PPARG	PPARG	IGF1

Figure 1B displays the PPI network with key hub genes, including CASP3, EGFR, ESR1, STAT3, HSP90AA1, SRC, ALB, PTEN, IGF1, and FN1, highlighting their central role in hepatocellular carcinoma progression and treatment.

**FIGURE 2:**
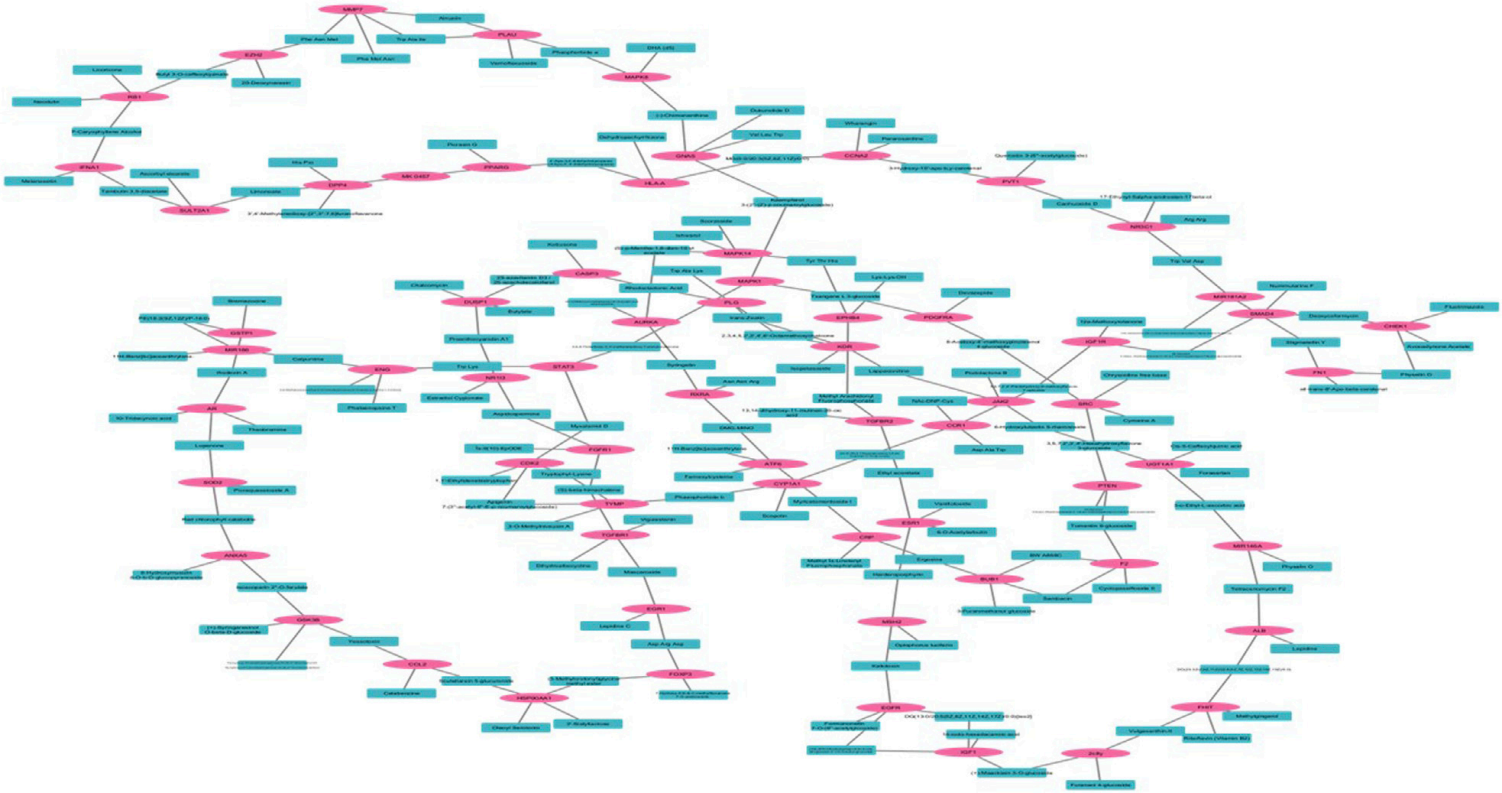
Compound and Gene Network whereas pink shows the targeted gene and blue color shows the compound.

A compound gene network was developed to visualize the complex relationships between genes and their corresponding biological functions in Figure 2. In this network, blue nodes represent biological compounds or functions, whereas pink nodes denote individual genes.

Among the identified hub genes, CASP3 and STAT3 were selected for detailed downstream analysis based on both biological significance and network topology. Functionally, STAT3 is a key oncogenic transcription factor involved in promoting cell proliferation, survival, and immune evasion in hepatocellular carcinoma (HCC), while CASP3 plays a central role in executing apoptosis—a process often suppressed during cancer progression.

Network analysis further supported their selection: both genes exhibited high degree centrality and betweenness within the compound-gene interaction network, suggesting pivotal roles in mediating compound-target relationships. Their dual involvement in tumor-promoting and tumor-suppressive pathways provided a compelling basis for their prioritization in virtual screening and molecular dynamics simulations, allowing evaluation of Alnusiin’s therapeutic potential against critical HCC-related mechanisms.

#### 3.2.2 GO and KEGG pathway enrichment analysis

**FIGURE 3:**
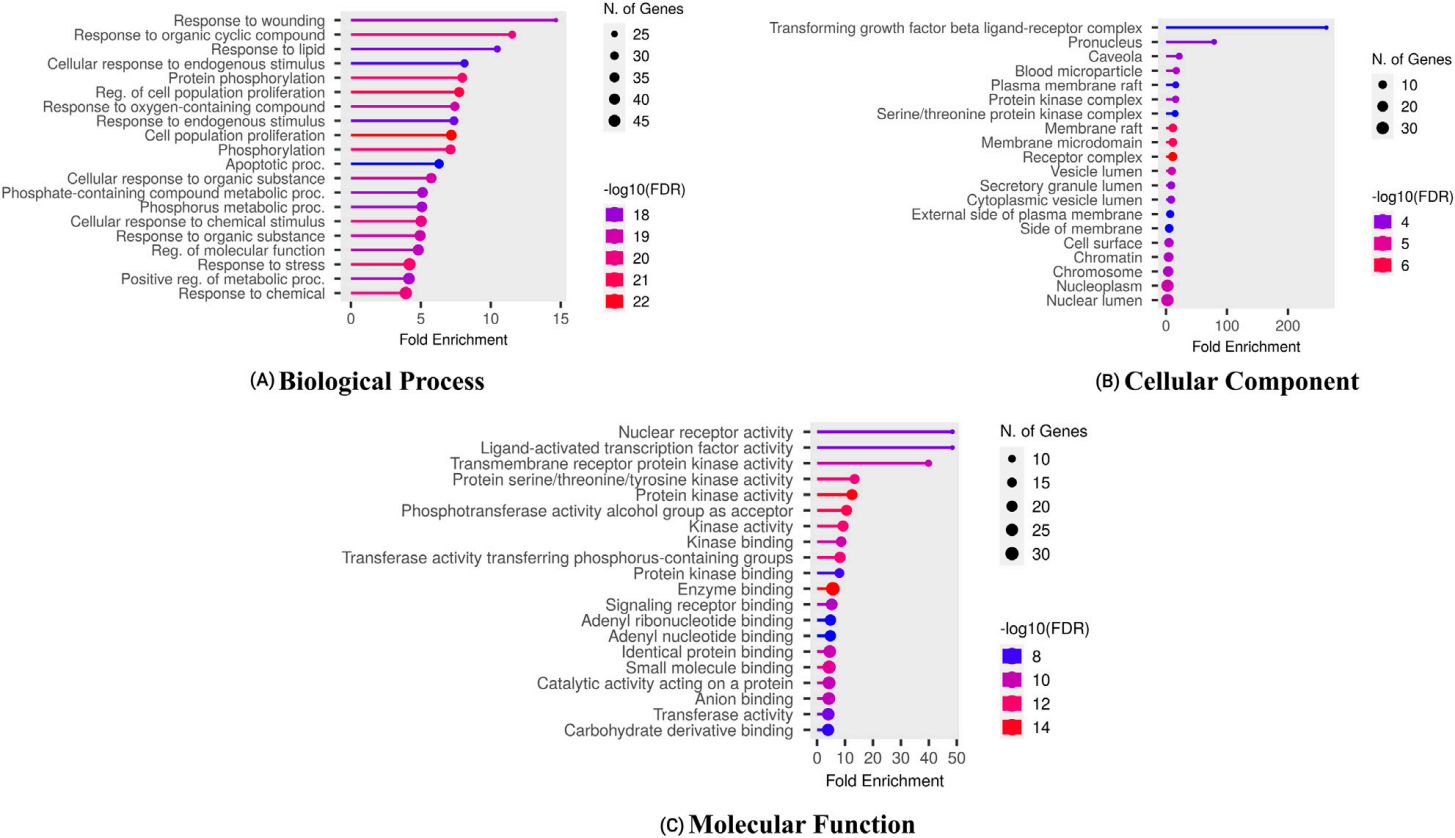
Gene ontology of proteins regulated by bioactives of *C. lancifolius* for **(A)** molecular function, **(B)** cellular components, and **(C)** biological process.

Enrichment analysis using the DAVID tool identified key GO terms and KEGG pathways associated with the overlapping target genes, with statistical significance set at p < 0.05. Figure 3 highlights the top ten enriched GO terms across Biological Processes (BP), Cellular Components (CC), and Molecular Functions (MF). Notable BP terms include cellular responses to peptides and reactive oxygen species metabolism. Significant CC terms involve blood microparticles and vesicle lumens, while enriched MF terms include ligand-activated transcription factor activity and insulin receptor binding. These results reveal functionally relevant pathways potentially involved in the pathophysiology of HCC.

**FIGURE 4:**
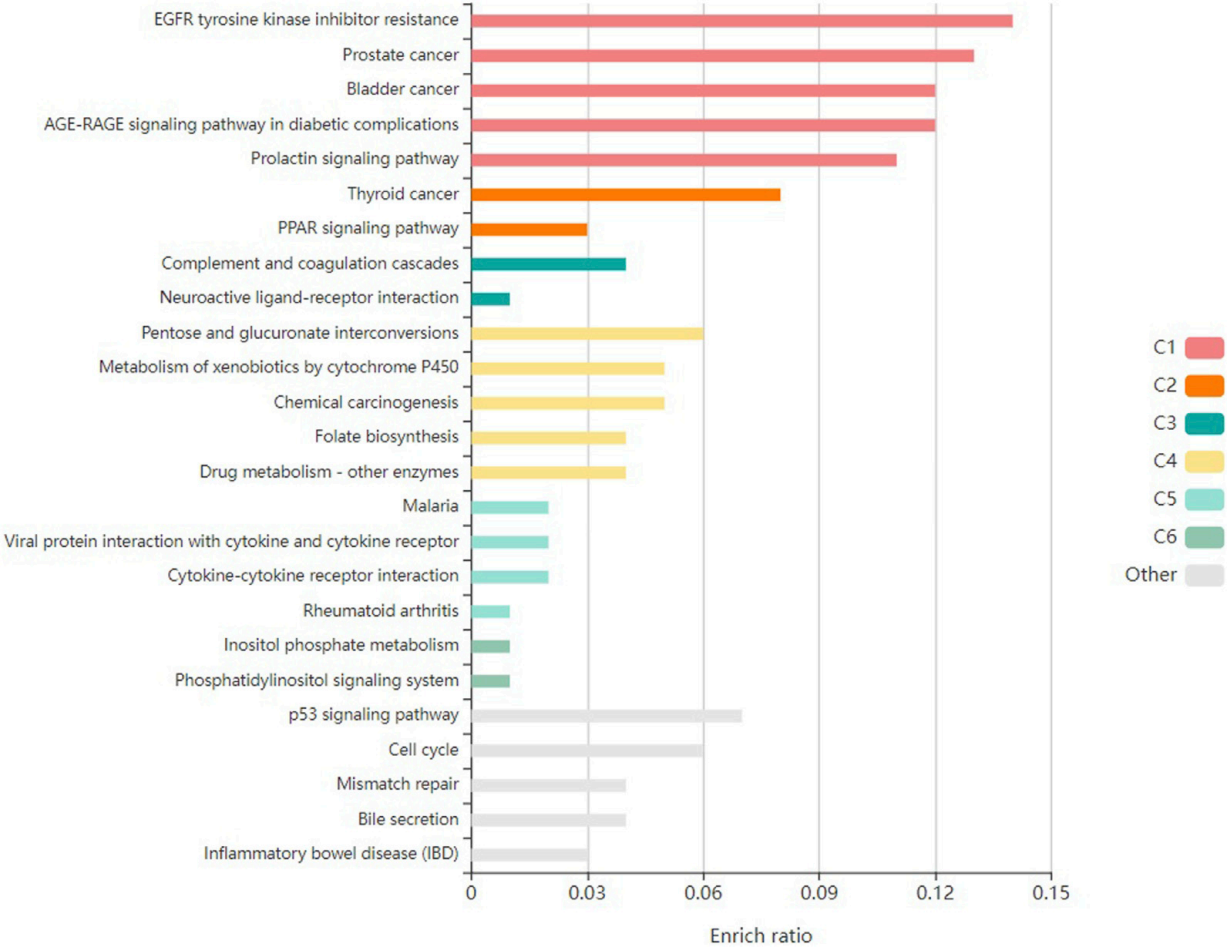
Kegg pathway enrichment analysis of top-targeted genes in *Conocarpus*.

Furthermore, the enrichment analysis revealed that the pathological processes of certain genes were significantly influenced by the top-targeted genes associated with KEGG pathways (Figure 4). The key targeted genes were linked to the following pathways: PPAR signaling, thyroid cancer, prostate cancer, prolactin signaling, and bladder cancer. Moreover, additional enriched pathways included chemical carcinogenesis, the p53 signaling pathway, and EGFR tyrosine kinase inhibitor resistance.

### 3.3 Virtual screening

#### 3.3.1 Virtual screening: identification of potential ligands from *C. lancifolius* to inhibit HCC

Virtual screening, utilizing molecular docking, is a highly effective computational tool for identifying potential lead compounds against specific targets. By applying this strategy, we identified compounds exhibiting strong binding affinities and specific interactions with key target proteins. Molecular docking analysis was performed to investigate the interactions between the bioactive phytochemicals of *C. lancifolius* and human protein targets implicated in HCC progression. During the docking analysis, multiple compounds exhibited strong binding affinities with the target proteins. Table 2 presents the highest binding affinities observed in the docking analysis, including CASP3-Alnusiin (11.512 kcal/mol), STAT3-Alnusiin (12.969 kcal/mol), CASP3-Yessotoxin (9.757 kcal/mol), CASP3-Ergosine (9.201 kcal/mol), STAT3–Apigenin 7-(3″acetyl-6″-E-p-coumaroyl glucoside) (11.046 kcal/mol), and STAT3–Dukunolide D (11.023 kcal/mol).

**TABLE 2:** Binding scores of top 3 phytochemicals with CASP3 and STAT3 proteins.

Sr.no.	Protein ID	Ligand Name	Binding energy	Contacting receptor residue
1	CASP3 (IGFW)	Alnusiin	11.512 kcal/mol	TRP 206 ARG 207 ASN 208 SER 209 ASP 211 TRP 214 GLN 217 PHE 247 GLU 248 SER 249 PHE 250 SER 251 PHE 252 ASP 253 PHE 256
2		Yessotoxin	9.757 kcal/mol	MET 61 HIS 121 GLY 122 GLU 123 PHE 128 CYS 13 THR 166 TYR 204 SER 205 TRP 206 ARG 207 ASN 208 SER 209 LYS 210 ASP 211 MET 222 GLN 225 TYR 226 LYS 242 GLU 246 PHE 247 GLU 248 SER 249 PHE 250 SER 251 PHE 256
3		Ergosine	9,201 kcal/mol	TYR 204 TRP 206 ARG 207 ASN 208 SER 209 LYS 210 TRP 214 SER 249 PHE 250 SER 251 PHE 252 ASP 253 PHE 256
1	STAT3 (6TLC)	Alnusiin	12.969 kcal/mol	GLU 357 LEU 358 GLN 361 LEU 362 LYS 363 GLU 444 VAL 445 TYR 446 GLN 448 GLY 449 LYS 282 GLU 357 LEU 358 GLN 361 VAL 445 TYR 446 HIS 447 GLN 448 GLY 449
2		Apigenin 7-(3”acetyl-6”-E-p-coumaroyl glucoside)	11.046 kcal/mol	GLN 279 LYS 282 LYS 283 GLU 286 GLU 357 LEU 358 GLN 361 VAL 445 TYR 446 HIS 447 GLN 448 GLY 449 GLU 357 LEU 358 TYR 360 GLN 361 LEU 362 VAL 393 GLU 444 VAL 445 TYR 446 GLN 448 GLY 449
3		Dukunolide D	11.023 kcal/mol	GLU 357 GLN 361 TYR 446 HIS 447 GLN 448 GLY 449 GLU 357 LEU 358 GLN 361 VAL 445 TYR 446 HIS 447 GLN 448 GLY 449

**FIGURE 5:**
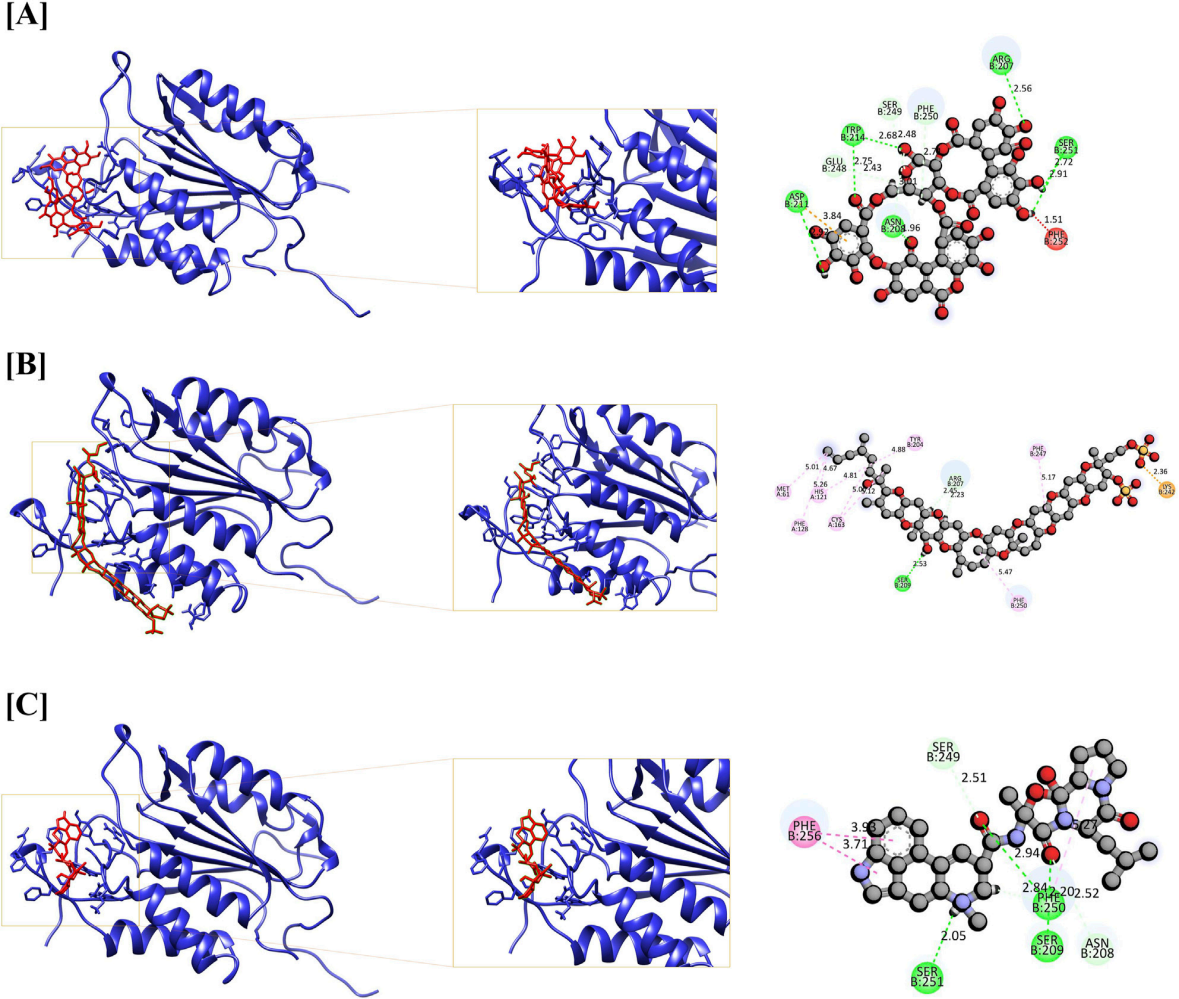
Molecular docking interaction of CASP3 with top three bioactive compounds. **(A)** Alnusiin, **(B)** Yessotoxin and **(C)** Ergosine. The figure illustrates the binding conformations of each compound within the STAT3 active site, highlighting key residues involved in hydrogen bonding and hydrophobic interactions that contribute to binding stability.

Additionally, the CASP3-Yessotoxin complex (9.757 kcal/mol) formed a single conventional hydrogen bond with SER209, along with seven alkyl and π-alkyl interactions involving MET61, HIS121, PHE128, CYS163, TYR204, PHE250, and PHE247. On the other hand, CASP3-Ergosine (9.201 kcal/mol) formed four conventional hydrogen bonds with SER251, SER209, and two PHE250 residues, as well as three carbon-hydrogen bonds with SER249, PHE250, and ASN208. Additionally, it exhibited two π-π stacked interactions with two PHE256 residues and one π-alkyl interaction with PHE250, as shown in Figure 5.

Apigenin 7-(3″acetyl-6″-E-p-coumaroyl glucoside) demonstrated a strong binding affinity for STAT3 (11.046 kcal/mol), forming four conventional hydrogen bonds (TYR446, GLU286, and two GLN361 residues), three carbon-hydrogen bonds (VAL445, GLN448, and LYS283), one amide-π stacked interaction (LYS282), and one π-alkyl interaction (VAL393). Similarly, STAT3–Dukunolide D (11.023 kcal/mol) formed four conventional hydrogen bonds (TYR446, GLN448, and two TYR446 residues), one carbon-hydrogen bond (GLN448), and one π-alkyl interaction (GLN448).

**FIGURE 6:**
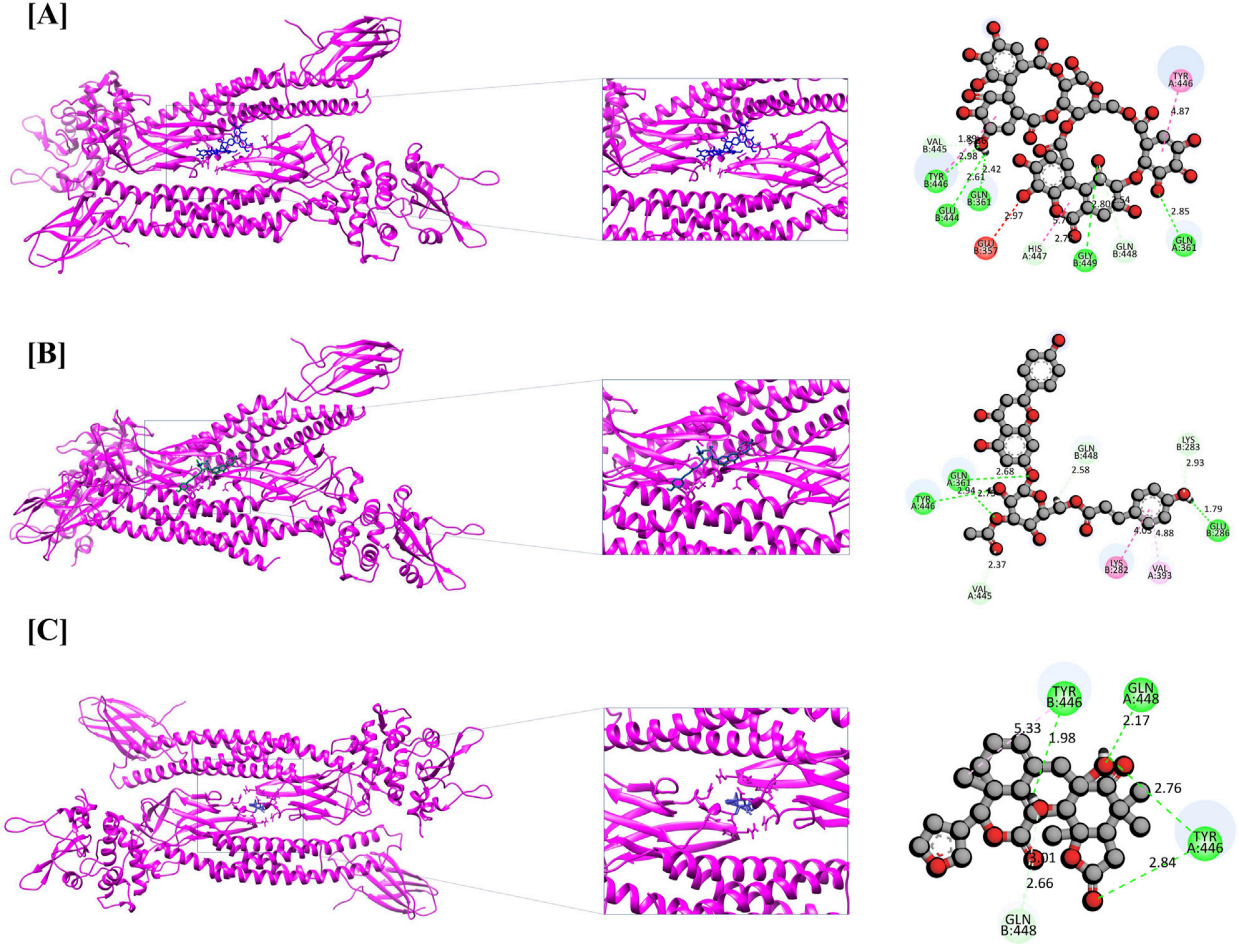
Molecular docking interaction of STAT3 with top three bioactive compounds. **(A)** Alnusiin, **(B)** Apigenin 7-(3″-acetyl-6″-E-p-coumaroyl glucoside), and **(C)** Dukunolide D. The figure illustrates the binding conformations of each compound within the STAT3 active site, highlighting key residues involved in hydrogen bonding and hydrophobic interactions that contribute to binding stability.

The compounds were found to interact with human target proteins implicated in HCC by binding at multiple active sites, as illustrated in Figure 6 and Table 2. Based on the docking scores, the CASP3-Alnusiin and STAT3-Alnusiin complexes were selected for further molecular dynamics simulation analysis to evaluate their stability and structural dynamics.

### 3.4 Molecular dynamic simulation

To evaluate the stability and conformational dynamics of key protein–ligand complexes, 100 ns molecular dynamics (MD) simulations were performed using the Desmond module in Schrödinger Maestro (Lakhani et al., 2024). The analysis specifically targeted CASP3-Alnusiin and STAT3-Alnusiin complexes, assessing their structural integrity in a solvated environment. Simulations were conducted at 300 K in a dodecahedral box with SPC water and neutralized using Na^+^ ions. After energy minimization and equilibration, the complexes were analyzed using RMSD, radius of gyration (Rg), RMSF, and hydrogen bond metrics. These parameters provided detailed insights into the dynamic stability and interaction profiles of the ligand-bound systems.

**FIGURE 7:**
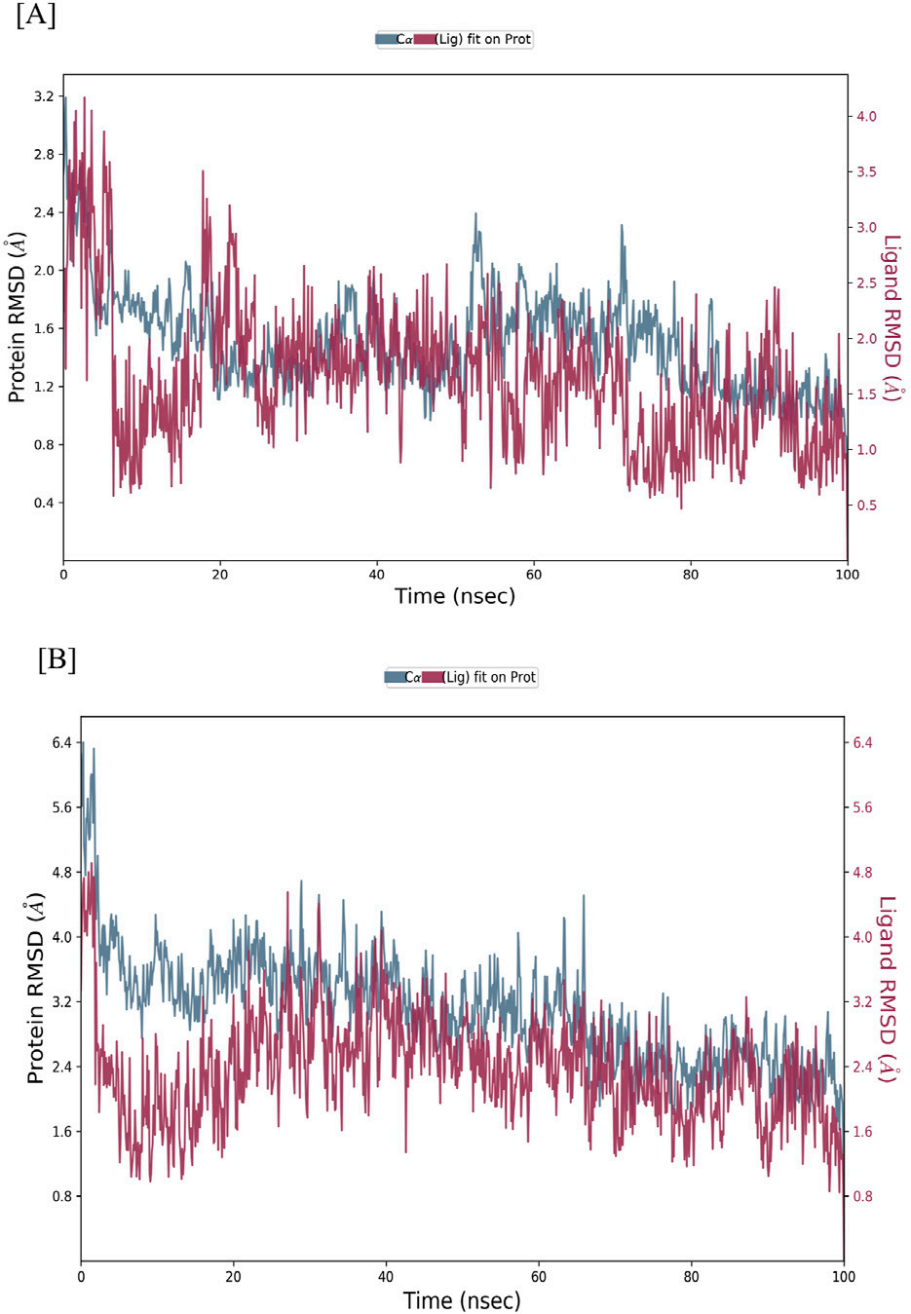
RMSD values of Alnussin with receptor proteins. **(A)** RMSD of the C-alpha atoms of CASP3 and Alnusiin; **(B)** RMSD of the Cα atoms of STAT3 and Alnusiin with time. The variation in RMSD of receptor protein is shown on the y-axis (on left) through time. The variation in RMSD of the ligand is shown on the y-axis (on right) through time.

To assess the structural stability of the CASP3-Alnusiin and STAT3-Alnusiin complexes, root mean square deviation (RMSD) analysis was conducted over the course of a 100 ns molecular dynamics simulation. This analysis provided insights into conformational stability and the equilibrium behavior of the ligand-bound protein systems, both of which are critical for evaluating therapeutic potential. For the CASP3-Alnusiin complex (Figure 7A), the RMSD profile indicated an initial adjustment phase (0–20 ns) followed by stabilization around 30 ns. The complex maintained an RMSD within ∼3 Å from 30 to 90 ns, suggesting a thermodynamically stable conformation and persistent ligand binding. The RMSD fluctuation of the CASP3-Alnusiin complex was significantly lower than that of the apo-CASP3 (p < 0.01), indicating that ligand binding stabilizes the protein structure. A minor dip at 65 ns did not compromise the overall structural integrity, confirming that Alnusiin formed a robust complex with CASP3. Similarly, the STAT3-Alnusiin complex (Figure 7B) reached equilibrium at approximately 40 ns and remained stable with an RMSD of ∼2.8 Å until 80 ns. The RMSD fluctuation of the STAT3-Alnusiin complex was also significantly lower than that of the apo-STAT3 (p < 0.01), further supporting the stabilizing effect of Alnusiin binding. Mild fluctuations observed thereafter did not exceed acceptable thresholds, further supporting the sustained interaction between STAT3 and Alnusiin.

**FIGURE 8:**
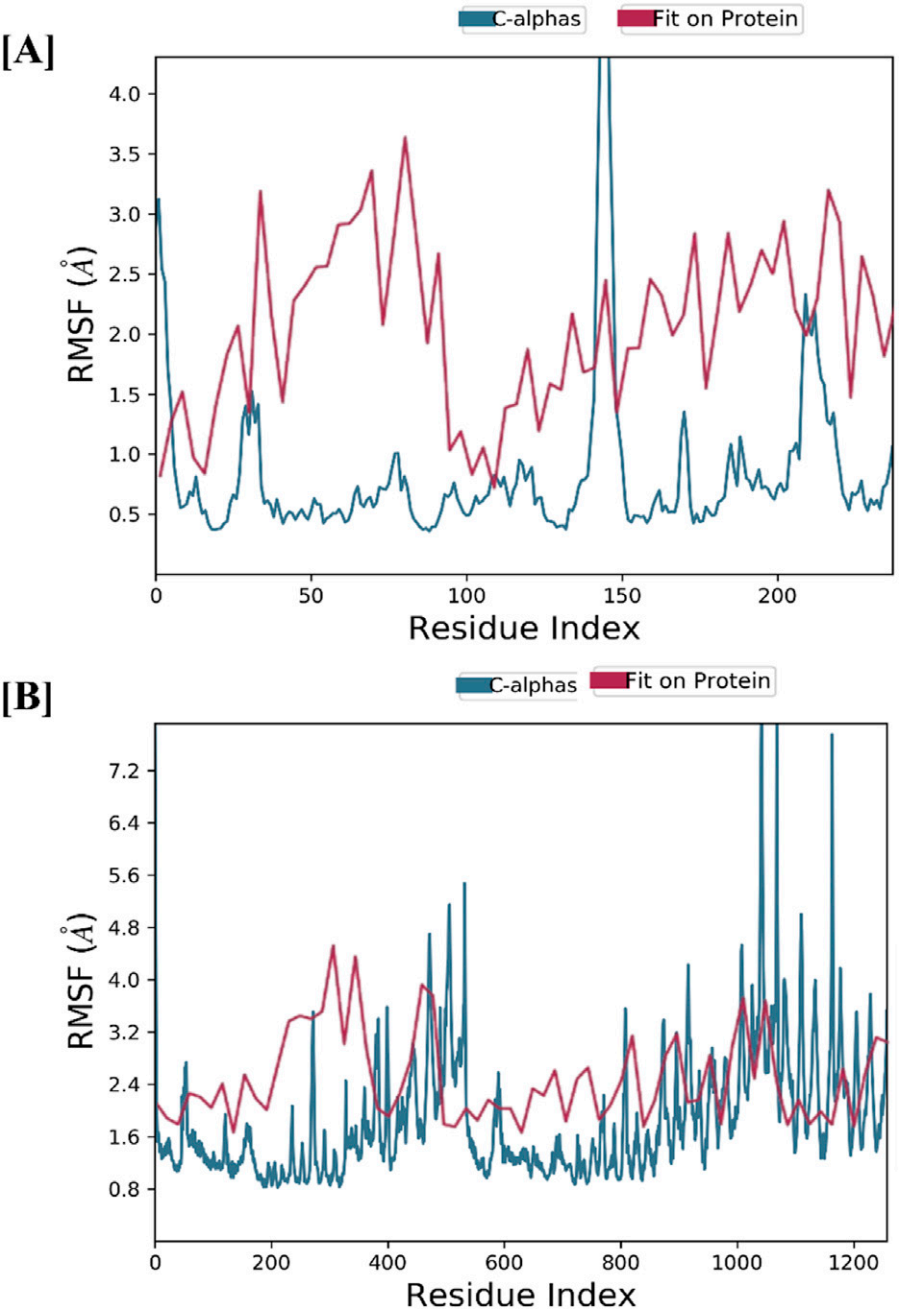
The plots of residue wise root mean square fluctuation (RMSF). **(A)** RMSF plot of CASP3 with Alnusiin and **(B)** RMSF plot of STAT3 with Alnusiin.

The structural stability of CASP3-Alnusiin and STAT3-Alnusiin complexes was evaluated over a 100 ns molecular dynamics simulation. Both complexes exhibited a consistent RMSD profile after an initial equilibration period, indicating stable ligand binding. For CASP3-Alnusiin (Figure 7A), RMSD stabilized at ∼3 Å from 30 to 90 ns, while STAT3-Alnusiin (Figure 7B) reached a plateau around 40 ns with RMSD values near 2.8 Å. Notably, the RMSD fluctuations of both complexes were significantly lower than their respective apo forms (CASP3 and STAT3) (p < 0.01), demonstrating enhanced structural rigidity upon Alnusiin binding. RMSF analysis (Figure 8A) revealed limited residue-level flexibility in both complexes. For the CASP3-Alnusiin complex, RMSF values ranged between 1.8 and 4.0 Å, with higher flexibility observed at the N- and C-terminal regions. In contrast, the binding site residues and secondary structural elements (α-helices and β-strands) exhibited minimal fluctuations, indicating enhanced rigidity compared to apo-CASP3. For the STAT3-Alnusiin complex, RMSF values ranged from 1.6 to 3.9 Å. Similar to CASP3, terminal and loop regions were more flexible, while core structural regions remained stable. Residues within the ligand-binding site showed reduced fluctuations compared to the apo-STAT3 protein, highlighting the stabilizing influence of Alnusiin.

**FIGURE 9:**
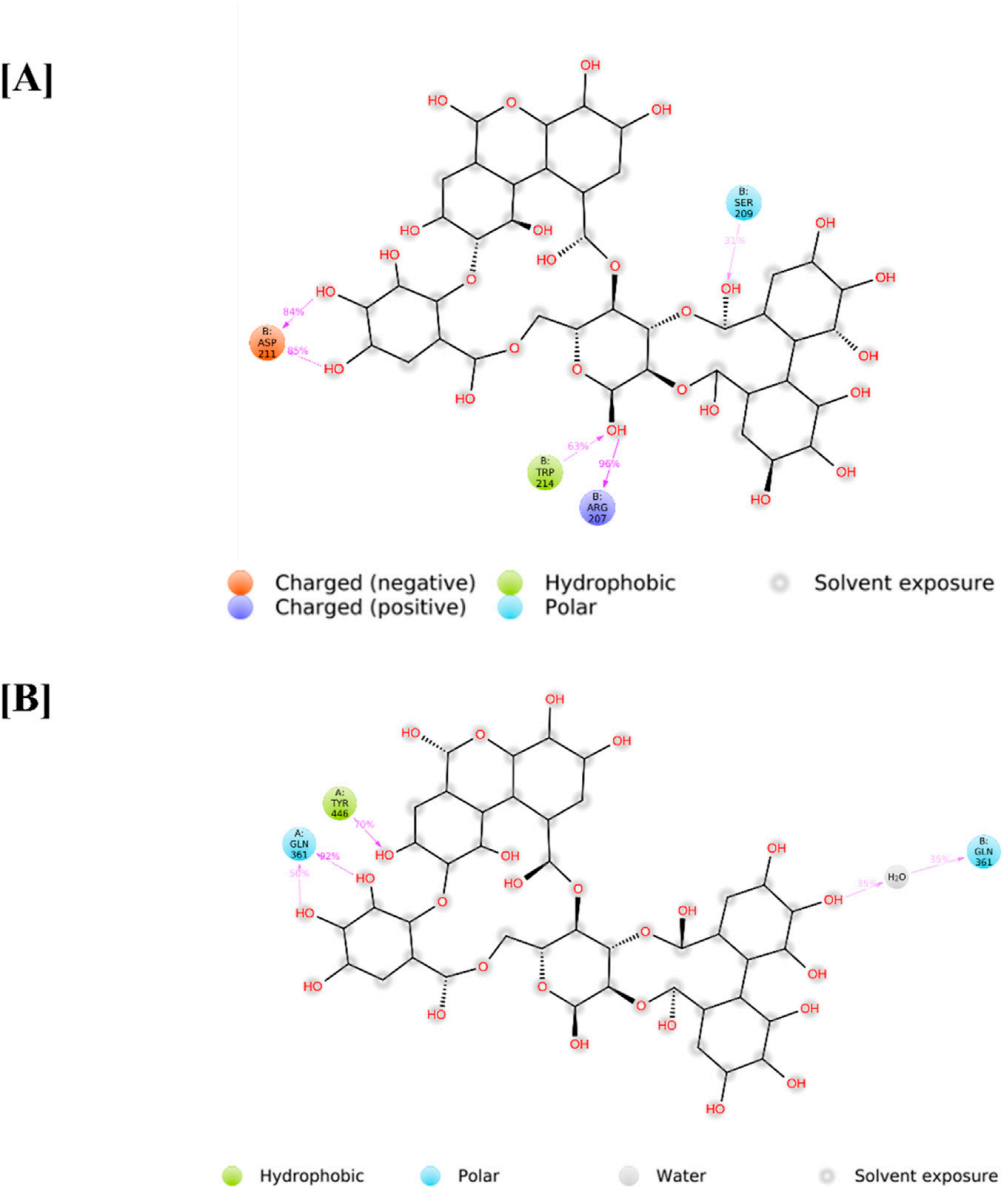
Detailed ligand intereactions with protein residues of **(A)** PDB -IGFW and **(B)** pDB - 6TLC.

**FIGURE 10:**
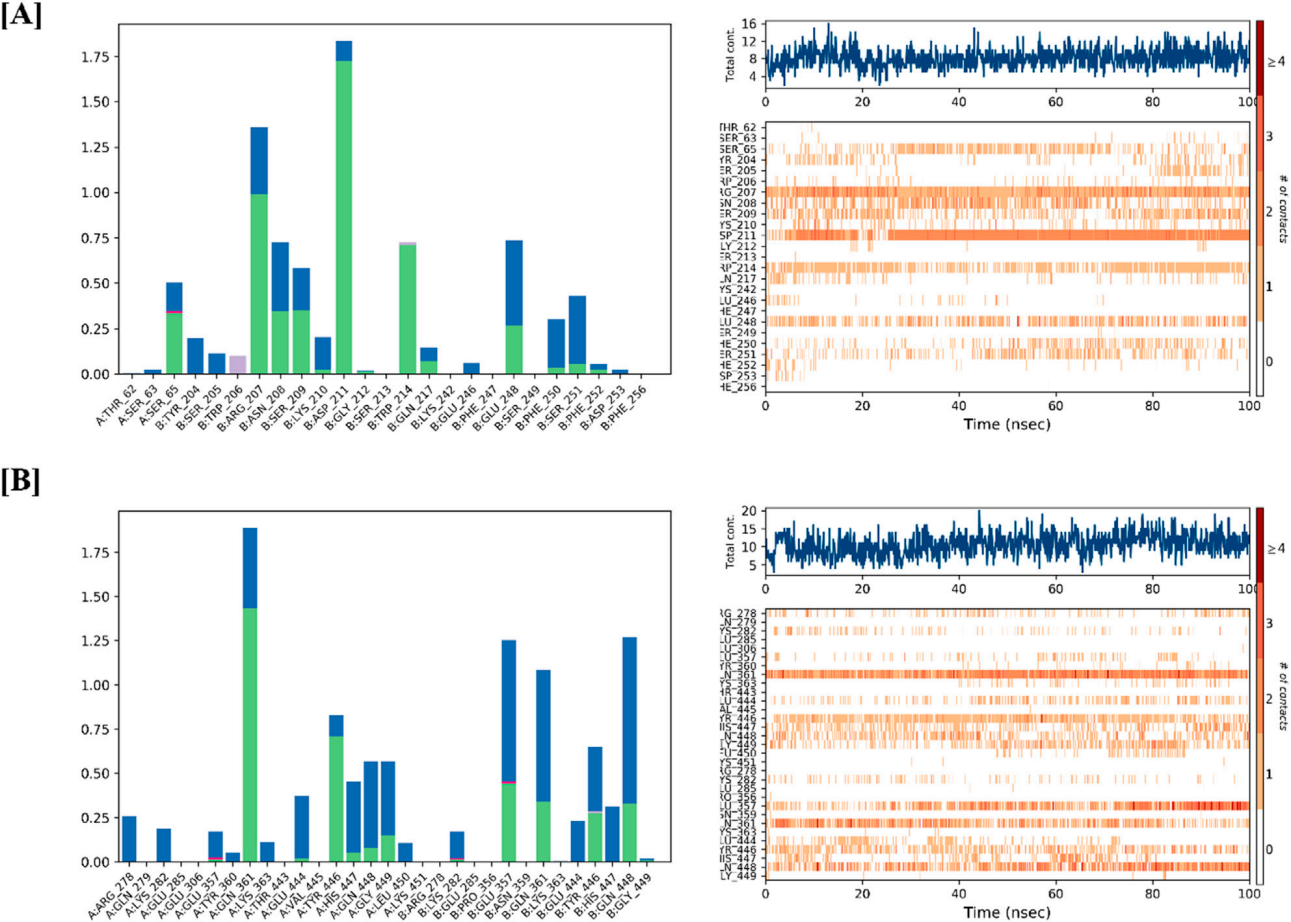
Protein-Ligand interaction profile of **(A)** CASP3-Alnussin and **(B)** STAT3-Alnusiin, interaction profile of crucial interacting amino acids, timeline representation of the interactions of amino acids.

Protein–ligand interactions observed during the simulation can be classified using the Simulation Interactions Diagram (SID) board provides a detailed breakdown of the subtypes within each interaction category into four main types: water-mediated bonds, hydrophobic interactions, ionic bonds, and hydrogen bonds (Figure 9). Assessing the total number of hydrogen bonds formed throughout the simulation helps evaluate the conformational stability of the protein–ligand complex. Detailed interaction profiling (Figures 10A,B) using Simulation Interaction Diagrams revealed stable hydrogen bonds, hydrophobic contacts, ionic interactions, and water bridges throughout the 100 ns trajectory. In the interaction pattern between the Alnusiin ligand and the CASP3 protein complex, hydrogen bonds with 13 key amino acid residues were observed viz., Ser65, Arg207, Asn208, Ser209, Lys210, Asp211, Gly212, Trp214, Gln217, Glu248, Phe250, Ser251, and Phe252 (Figure 10A). Among these, Asp211 exhibited the highest hydrogen bond interaction fraction (1.75), followed by Arg207 (1.0), indicating their significant contribution to complex stability. The hydrogen bonds were stable throughout the 100 ns simulation, with the CASP3-Alnusiin complex. For the highest interaction fractions of 1.0 and 0.6, respectively. The average number of hydrogen bonds in this complex was 3.8, while the apo-STAT3 protein showed minimal hydrogen bond formation (average 0.9). These results clearly indicate that ligand binding enhances the number and stability of hydrogen bond interactions, contributing to the conformational stabilization of both CASP3 and STAT3 during the molecular dynamics simulation.

## 4 Discussion

Hepatocellular carcinoma (HCC) is a major global health concern characterized by complex etiologist, high incidence rates, and significant mortality. It involves multiple genetic, proteomic, and molecular pathway alterations that contribute to cancer progression and cancer-related deaths (Sung et al., 2021). Due to the increasing prevalence of HCC and the limited efficacy of current treatments, survival rates for HCC patients remain unsatisfactory. There is an urgent need for alternative therapeutic strategies that offer greater efficacy with reduced toxicity. Traditional medicinal systems such as Siddha and Ayurveda, along with indigenous knowledge, have long been utilized for the treatment of various clinical conditions (Meenatchisundaram et al., 2009). Additionally, research supports the successful application of bioactive compounds from medicinal plants as alternative therapeutic agents (Harvey, 2008).

*Conocarpus lancifolius* is a medicinally important species within the *Conocarpus* genus, belonging to the Combretaceae family (Ali et al., 2013). Several studies have demonstrated that the *Conocarpus* genus possesses a range of pharmacological properties, including antiprotozoal, leishmanicidal, antibacterial, and anticancer activities (Saadullah et al., 2024). LC-MS QTOF analysis of *C. lancifolius* extracts revealed the presence of numerous bioactive compounds, including flavonoids, fatty acids, and phenolic compounds, many of which have been isolated from other plants with documented pharmacological properties. Several of these compounds have exhibited antidiabetic effects in both *in vitro* and *in vivo* studies, with notable implications for HCC treatment (Alam et al., 2016). The findings from LC-MS QTOF analysis further confirm that *C. lancifolius* contains phytochemicals with significant anti-HCC potentia (Amin et al., 2017). Accordingly, all active compounds identified were subjected to *in silico* molecular docking analysis to elucidate their mechanisms of action as potential inhibitors of hepatocellular carcinoma (HCC).

The study employed network pharmacology to identify 65 potential targets associated with hepatocellular carcinoma. Pathway enrichment analysis provided insights into their biological significance, revealing the involvement of these targets in cellular processes, biological regulation, injury response, and cell proliferation. Gene Ontology (GO) analysis identified key cellular components and molecular functions linked to HCC progression. Additionally, pathway analysis highlighted significant enrichment in the PPAR signaling pathway, thyroid cancer, prostate cancer, and bladder cancer pathways. Through pharmacology network analysis, the study identified key compound-target interactions relevant to HCC. The ten most significant hub nodes identified were CASP3, EGFR, ESR1, HSP90AA1, SRC, ALB, PTEN, IGF1, FN1, and STAT3. Further analysis revealed that CASP3 and STAT3 were strongly associated with poor overall survival outcomes in HCC patients. Molecular docking validated compound-target interactions, demonstrating that Alnusiin, Yessotoxin, Ergosine, Apigenin 7-(3”acetyl-6”-E-p-coumaroyl glucoside), and Dukunolide D exhibited binding affinities within the acceptable range. To further confirm these findings, molecular dynamics (MD) simulations were conducted for 100 ns, providing additional validation of Alnusiin’s binding stability and effectiveness. Simulated validation assays demonstrated promising results, with stable root mean square deviation (RMSD) values for Alnusiin-bound CASP3 and STAT3, indicating well-matched protein-ligand interactions.

Previous research has shown that Yessotoxin increases intracellular calcium levels, triggering apoptosis in human hepatocellular carcinoma cells. This process can be inhibited by calcium ion chelation or the use of nifedipine. In HCC-induced rats, caspase-3 mRNA expression was reduced; however, curcumin administration significantly upregulated caspase-3 expression, demonstrating its apoptosis-inducing effects (Abouzied et al., 2015). Cancer cells often evade apoptosis by downregulating caspase-3 expression, leading to increased survival and resistance to chemotherapy. This resistance is frequently associated with genetic and molecular alterations affecting caspase-3 (Kolenko et al., 1999). As a key effector caspase, caspase-3 plays a crucial role in the execution phase of apoptosis. The active, cleaved form of caspase-3 is responsible for promoting apoptotic cell death (Medina et al., 1997). A previous study reported that caspase-3 modulates the movement of SREBP2 during the development of acquired drug resistance in HCC. Specifically, apoptosis was not induced in HCC cells transfected with SREBP2-D468A when caspase-3 was inhibited (Mok et al., 2022).

STAT3, a transcription factor, is recognized as a promising therapeutic target for hepatocellular carcinoma due to its pivotal role in oncogenesis. Initially, STAT3 was identified as a regulator of acute-phase gene expression in response to inflammatory stimuli (Zhong et al., 1994). STAT3 is closely associated with inflammation and oncogenic signaling, with its phosphorylation at Y705 frequently observed in HCC cases linked to poor prognosis. Thus, STAT3 has emerged as a potential target for HCC therapy and prevention (Lee and Cheung, 2019). STAT3 inhibitors function by blocking STAT3 activation in tumor cells and tumor-associated macrophages, thereby restoring antitumor immune responses and enhancing tumor suppression (Mano et al., 2013).

Post-docking analysis confirmed that 12 CLAE-derived compounds, including ellagic acid, exhibited high binding affinities to the target protein hAChE. Docking scores ranged from −7.848 to −15.180 kcal/mol, comparable to the FDA-approved drug galantamine, which exhibited a score of −9.742 kcal/mol (Khurm et al., 2023). Furthermore, an extensive literature review identified five additional proteins (NF-kB, TNF-α, CASP3, BCL-2, and BCL-XL) as key regulators of distinct signaling pathways in HepG2 cells. These proteins were subsequently selected for molecular docking investigations (Saadullah et al., 2021). A previous study conducted by (Siddiqui et al., 2021) investigated the molecular docking interactions of eight phytochemicals from Moringa oleifera fruit with caspase-3. Benzyl glucosinolate exhibited the strongest binding affinity (−8.4 kcal/mol), forming interactions with key residues within the caspase-3 active site, including Tyr204, Trp206, Thr62, Arg207, Phe250, and Phe265. In a similar manner, Suganya and Anuradha (2019), Similarly, Suganya and Anuradha (2019) conducted molecular docking studies on astaxanthin and sorafenib with multiple apoptotic proteins involved in HCC (Suganya and Anuradha, 2019). Additionally, docking results demonstrated that the STAT3-SH2 inhibitor N4 interacts with STAT3 at LYS591, SER636, VAL637, and GLU638, thereby inhibiting its function (Chen et al., 2023).

This study identifies CASP3 and STAT3 as promising therapeutic targets for hepatocellular carcinoma (HCC) using a network pharmacology approach. Bioactive compounds from *C. lancifolius* have been shown to modulate multiple molecular mechanisms involved in HCC pathogenesis. Among these, Alnusiin emerged as a potent anti-HCC agent by effectively inhibiting CASP3 and STAT3. While the computational outcomes offer compelling insights, experimental validation remains crucial to establish the therapeutic relevance of Alnusiin in hepatocellular carcinoma (HCC). Planned *in vitro* assays using widely accepted HCC cell lines (e.g., HepG2, Huh7, SNU-449) will investigate cytotoxicity, apoptotic induction, and caspase-3/7 activation via MTT, Annexin V/PI, and Caspase-Glo assays. Further, gene and protein expression of key targets including CASP3, STAT3, BCL-2, and BAX will be assessed through qRT-PCR and Western blotting to confirm mechanistic activity.

To explore functional dynamics, luciferase reporter assays will be used to track STAT3 inhibition, alongside ROS assays to examine oxidative stress involvement. Should *in vitro* results prove promising, *in vivo* studies will be undertaken in xenograft or orthotopic HCC mouse models to evaluate tumour suppression, pharmacokinetics, and potential toxicity. Histological and immunohistochemical analyses will support findings by identifying expression of apoptotic markers such as cleaved caspase-3 and phosphorylated STAT3 in liver tissues. This stepwise validation approach will provide robust evidence to support the progression of Alnusiin as a viable, plant-derived candidate for future preclinical development in HCC therapy.

## 5 Conclusion

This study comprehensively elucidated the relationship between the bioactive compounds of *C. lancifolius* identified through LC-MS QTOF analysis and hepatocellular carcinoma (HCC) at the molecular level. The findings indicate that *C. lancifolius* possesses significant potential as an anti-HCC agent for pharmaceutical development. A total of 154 bioactive compounds and their 65 putative protein targets were utilized to construct a Protein-Protein Interaction (PPI) network, providing insights into key molecular mechanisms. Additionally, Gene Ontology (GO) and KEGG pathway enrichment analyses were performed to further characterize the biological significance of these targets. Molecular docking experiments revealed that the principal bioactive compounds of *C. lancifolius* exhibited strong binding affinities toward the key HCC-related targets CASP3 and STAT3. The structural stability of the Alnusiin-phytochemical complexes was further validated through molecular dynamics (MD) simulations. These simulations were analyzed using root mean square deviation (RMSD), root mean square fluctuation (RMSF), and hydrogen bond (H-bond) analyses, confirming that all selected compounds maintained structural stability over the 100 ns MD simulation period. This study identified three major phytochemicals Alnusiin, Yessotoxin, and Ergosine that demonstrated significant binding affinities with HCC-related targets. The results suggest that compounds derived from *C. lancifolius*, particularly Alnusiin, may serve as potential inhibitors of CASP3 and STAT3 proteins in hepatocellular carcinoma (HCC) and could be explored for the development of improved anti-HCC therapeutics. However, further experimental validation, including *in vivo* studies and clinical trials, is essential to confirm the efficacy and therapeutic potential of these compounds for HCC treatment.

## Data Availability

The original contributions presented in the study are included in the article/Supplementary Material, further inquiries can be directed to the corresponding author.

## Abbreviations

Conocarpus lancifolius, C. lancifolius; MDS, Molecular dynamic simulation’; CASP3, Caspase 3; STAT3, signal transducer and activator of transcription 3; HCC, Hepatocellular carcinoma; BP, Biological Process; CC, Cellular Component; MF, Molecular Function; MMGBSA, Molecular Mechanics Generalized Born Surface Area; RMSD, Root Mean Square Deviation; RMSF, Root Mean Square Fluctuation.
