# Supplementary material for: Decoding the therapeutic mechanism of Conocarpus lancifolius in hepatocellular carcinoma: network pharmacology, molecular docking, and LC-MS QTOF insights
Source: Front Pharmacol. 2025 Jun 17;16:1582374. doi: 10.3389/fphar.2025.1582374 (PMC12209259; doi:10.3389/fphar.2025.1582374)
Supplement: Supplementary file 1 [file Table1.docx]

**Table 1: List of compounds**

| Sr.no | Compound Name | Sr.no | Compound Name |
| --- | --- | --- | --- |
| 1 | Sambacin | 78 | Lappaconitine |
| 2 | 2,3,4,5,2',3',4',6'-Octamethoxychalcone | 79 | Myxalamid D |
| 3 | Apigenin 7-(3''-acetyl-6''-E-p-coumaroylglucoside) | 80 | Phe Asn Met |
| 4 | Alnusiin | 81 | Kalkitoxin |
| 5 | (15a,20R)-Dihydroxypregn-4-en-3-one 20-[glucosyl-(1->4)-6-acetyl-glucoside] | 82 | Yessotoxin |
| 6 | Isoscoparin 2''-O-ferulate | 83 | 3-Hydroxy-10'-apo-b,y-carotenal |
| 7 | MG(0:0/20:3(5Z,8Z,11Z)/0:0) | 84 | Myricatomentoside I |
| 8 | DMG-MINO | 85 | 25-azavitamin D3 / 25-azacholecalciferol |
| 9 | Proanthocyanidin A1 | 86 | (3S,7E,9R)-4,7-Megastigmadiene-3,9-diol 9-[apiosyl-(1->6)-glucoside] |
| 10 | 5,6,7,3',4'-Pentahydroxy-8-methoxyflavone 7-apioside | 87 | (-)-Chimonanthine |
| 11 | Kaempferol 3-(2''-(Z)-p-coumaroylglucoside) | 88 | Vulgaxanthin-II |
| 12 | DG(20:3(5Z,8Z,11Z)/22:6(4Z,7Z,10Z,13Z,16Z,19Z)/0:0) | 89 | Lupenone |
| 13 | rhodexin A | 90 | MK 0457 |
| 14 | Limonoate | 91 | (S)-p-Mentha-1,8-dien-10-yl acetate |
| 15 | Tyr Thr His | 92 | (S)-beta-himachalene |
| 16 | Mascaroside | 93 | Deoxycoformycin |
| 17 | Physalin D | 94 | 8-Acetoxy-4'-methoxypinoresinol 4-glucoside |
| 18 | 3,5,7,2',3',4'-Hexahydroxyflavone 3-glucoside | 95 | 3-o-Ethyl-L-ascorbic acid |
| 19 | 6-Hydroxyluteolin 5-rhamnoside | 96 | Calpurnine |
| 20 | Trp Lys | 97 | Î²-Caryophyllene Alcohol |
| 21 | Butyl 3-O-caffeoylquinate | 98 | Ethyl aconitate |
| 22 | Harderoporphyrin | 99 | (3-Methylcrotonyl)glycine methyl ester |
| 23 | Scutellarein 5-glucuronide | 100 | Trp Val Asp |
| 24 | Caohuoside D | 101 | BW A868C |
| 25 | Ergosine | 102 | trans-Zeatin |
| 26 | Rhodocladonic Acid | 103 | Tryptophyl-Lysine |
| 27 | Phaeophorbide b | 104 | Trp Ala Ile |
| 28 | Pheophorbide a | 105 | DG(13:0/20:5(5Z,8Z,11Z,14Z,17Z)/0:0)[iso2] |
| 29 | (+)-Maackiain 3-O-glucoside | 106 | (+)-Syringaresinol O-beta-D-glucoside |
| 30 | Red chlorophyll catabolite | 107 | Pararosaniline |
| 31 | 4'-Apo-3,4-didehydrolycopene/ (4-Apo-3',4'-didehydrolycopene) | 108 | 11H-Benz[bc]aceanthrylene |
| 32 | Syringetin | 109 | Chalcomycin |
| 33 | Aspidospermine | 110 | Asp Ala Trp |
| 34 | (S)-Nerolidol 3-O-[a-L-rhamnopyranosyl-(1->4)-a-L-rhamnopyranosyl-(1->6)-b-D-glucopyranoside] | 111 | Val Leu Trp |
| 35 | Tsangane L 3-glucoside | 112 | Podolactone B |
| 36 | Tetracenomycin F2 | 113 | 3,5,8-Trimethoxy-3',4'-methylenedioxy-7-prenyloxyflavone |
| 37 | PE(18:2(9Z,12Z)/P-16:0) | 114 | 20-Deoxynarasin |
| 38 | Tambulin 3,5-diacetate | 115 | Oplophorus luciferin |
| 39 | Methyl Arachidonyl Fluorophosphonate | 116 | Celabenzine |
| 40 | Asp Arg Asp | 117 | Quercetin 3-(6''-acetylglucoside) |
| 41 | Stigmatellin Y | 118 | Methyl Î±-Linolenyl Fluorophosphonate |
| 42 | Tomentin 6-glucoside | 119 | Kobusone |
| 43 | Riboflavin (Vitamin B2) | 120 | Scopolin |
| 44 | Theobromine | 121 | DHA (d5) |
| 45 | His Pro | 122 | Furaneol 4-glucoside |
| 46 | Ishwarol | 123 | Picraquassioside A |
| 47 | Viguiestenin | 124 | Picrasin G |
| 48 | Fluotrimazole | 125 | N1-(2-Methoxy-4-methylbenzyl)-n2-(2-(pyridin-2-yl) ethyl)oxalamide |
| 49 | Chrysoidine free base | 126 | Î±-9(10)-EpODE |
| 50 | Cis-5-Caffeoylquinic acid | 127 | Nummularine F |
| 51 | 3-(2-Methylpropanoyloxy)-8-(3-methylbutanoyloxy)-9,10-epoxy-p-mentha-1,3,5-triene | 128 | Devazepide |
| 52 | Neodulin | 129 | Physalin O |
| 53 | 6-O-Acetylarbutin | 130 | 11H-Benz[bc]aceanthrylene. |
| 54 | Oleoyl Serotonin | 131 | Melanoxetin |
| 55 | 17-Ethynyl-5alpha-androstan-17beta-ol | 132 | 13,14-dihydroxy-11-mulinen-20-oic acid |
| 56 | 3-Furanmethanol glucoside | 133 | 7-Hydroxy-6,8-di-C-methylflavanone 7-O-arabinoside. |
| 57 | Trp Ala Lys | 134 | 24S-methylcholest-22E-en-3beta,4beta,5alpha,6alpha,8beta,14alpha,15alpha,25,28-nonol |
| 58 | 3-O-Methylniveusin A | 135 | Cyclopassifloside II |
| 59 | Vernoflexuoside | 136 | Isopetasoside |
| 60 | 16-iodo-hexadecanoic acid | 137 | 1,1'-Ethylidenebistryptophan |
| 61 | 6-Hydroxymusizin 8-O-b-D-glucopyranoside | 138 | Phe Met Asn |
| 62 | Dehydropachyrrhizone | 139 | Formononetin 7-O-(6''-acetylglcoside) |
| 63 | Asn Asn Arg | 140 | 1Î±-hydroxy-24-(dimethylphosphoryl)-25,26,27-trinorvitamin D3 / 1Î±-hydroxy-24-(dimethylphosphoryl)-25,26,27-trinorcholecalciferol |
| 64 | Estradiol Cypionate | 141 | Wharangin |
| 65 | 12a-Methoxyrotenone | 142 | Farnesylcysteine |
| 66 | 3,5,8-Trimethoxy-3',4'-methylenedioxy-7-prenyloxyflavone | 143 | Butylate |
| 67 | Lepidine | 144 | NAc-DNP-Cys |
| 68 | Bremazocine | 145 | Dukunolide D |
| 69 | Ascorbyl stearate | 146 | Methylgingerol |
| 70 | Lys-Lys-OH | 147 | 10-Tridecynoic acid |
| 71 | Lepidine C | 148 | 3',4'-Methylenedioxy-[2'',3'':7,8]furanoflavanone |
| 72 | all-trans-8'-Apo-beta-carotenal | 149 | Scorzoside |
| 73 | (S)-Nerolidol 3-O-[a-L-Rhamnopyranosyl-(1->4)-a-L-rhamnopyranosyl-(1->2)-b-D-glucopyranoside] | 150 | Dihydroalbocycline |
| 74 | Licoricone | 151 | Avocadynone Acetate |
| 75 | Vanilloloside | 152 | Cyrneine A |
| 76 | 3'-Sialyllactose | 153 | Forasartan |
| 77 | Arg Arg | 154 | Phalaenopsine T |
